# Dl-NBP (Dl-3-N-Butylphthalide) Treatment Promotes Neurological Functional Recovery Accompanied by the Upregulation of White Matter Integrity and HIF-1α/VEGF/Notch/Dll4 Expression

**DOI:** 10.3389/fphar.2019.01595

**Published:** 2020-01-24

**Authors:** Yanping Wang, Yufei Shen, Ziyun Liu, Jingxia Gu, Congying Xu, Shuxia Qian, Xiaoling Zhang, Beiqun Zhou, Yuhua Jin, Yanyun Sun

**Affiliations:** ^1^Department of Neurology, The Second Affiliated Hospital of Jiaxing City, Jiaxing, China; ^2^Department of Neurology, Bengbu Medical College, Bengbu, China; ^3^Jiangsu Key Laboratory of Neuropsychiatric Diseases Research and Institute of Neuroscience, The Second Affiliated Hospital of Soochow University, Suzhou, China

**Keywords:** dl-NBP, neurological functional recovery, white matter, HIF-1α-VEGF-Notch-Dll4, mice

## Abstract

Dl-3-n-butylphthalide (dl-NBP) was approved by the FDA of China for the treatment of acute ischemic stroke. Dl-NBP has been shown to promote neurological functional recovery and enhance white matter integrity using an endothelin-1-induced focal permanent cerebral ischemia model, which could mimic those patients who have no opportunity to receive either tissue plasminogen activator (tPA) thrombolysis or endovascular therapy. However, it is not clear whether dl-NBP could promote neurological functional recovery in a focal transient cerebral ischemia model, which could mimic those patients who have the opportunity to receive either tPA thrombolysis or endovascular therapy. In this study, using a model of middle cerebral artery occlusion in mice, we aim to explore the effect of two-week dl-NBP treatment on neurological functional recovery after ischemic stroke as well as its underlying mechanism. Our results showed that dl-NBP treatment promoted functional recovery assessed by neurological scores and an adhesive remove test, and this improved the integrity of white matter after 60-min ischemia and 14-day reperfusion. In addition, dl-NBP increased the number of RECA-1 positive vessels and enhanced the expression of the tight junction protein occludin. More importantly, dl-NBP also promoted the expression of hypoxia-induced factor-1α, the vascular endothelial growth factor, Notch, and delta-like ligand 4. In conclusion, our study provides evidence that dl-NBP treatment could also promote functional recovery after focal transient ischemia stroke, and this recovery is associated with upregulated white matter integrity, microvessels, and the tight junction protein occludin. Our results suggested that, in future, dl-NBP may also be applied in clinic to promote functional recovery during the later phase of focal transient ischemic stroke.

## Introduction

Stroke is a leading cause of both morbidity and mortality all over the world. The only Food and Drug Administration (FDA)-approved drug to treat acute ischemic stroke is recombinant tissue plasminogen activator (rtPA). However, only 2–5% of stroke patients received rtPA treatment due to the narrow therapeutic time window and possible side effects of hemorrhage transformation (Wang et al., 2011; Wei et al., 2017). It is therefore urgent to figure out alternative compounds to promote functional recovery during the late phase of ischemic stroke.

The seed of *Apium Graveolens* Linn has a long history of treating hypertension, epilepsy, vertigo, and headache in China, and its active component, l-3-n-butylphthalide (l-NBP), was successfully isolated in the late 1970s. In 1980, the racemic compound dl-3-n-butylphthalide (dl-NBP) was synthesized, and it resolved the problem of limited resources from plant extracts (Huang et al., 2018). Dl-NBP was approved by the FDA of China for the treatment of ischemic stroke in 2002 (Wang et al., 2018), and it has been approved by the FDA of the USA to undergo a phase II trial for the treatment of ischemic stroke (Cheng et al., 2019). It is currently used in the acute phase of ischemic stroke in clinic and is a promising therapeutic agent for ischemic stroke (Wang et al., 2018). However, it has not been approved for use in the late phase of ischemic stroke as the effect is not confirmed and the mechanism is not clear.

Although l-NBP has been shown to promote functional recovery by promoting neurogenesis and neuroplasticity after transient focal cerebral ischemic stroke in rats (Yang et al., 2015), there are only limited resources from plant extracts, and it is dl-NBP, not l-NBP, that is currently used in clinic in China. Using an endothelin-1-induced focal permanent stroke model that could mimic those patients who have no opportunity to receive either tPA thrombolysis or endovascular therapy; Sun et al., showed that dl-NBP promoted neuroplasticity and motor recovery of rats (Sun et al., 2017b). However, it is not clear whether dl-NBP could promote functional recovery in a transient focal ischemic stroke model that could mimic those patients who have the opportunity to receive either tPA thrombolysis or endovascular therapy.

White matter damage accounts for half of the infarct volume (Wang et al., 2017). Destruction of white matter integrity is an important pathological basis for neurological impairment (Wang et al., 2017), and the blood–brain barrier (BBB) integrity is one of the key factors in the pathological damage of white matter hyperintensities (Li et al., 2017). Increasing the white matter integrity (Etherton et al., 2017) and increasing the integrity of BBB (Jiang et al., 2017) contribute to the neurological functional recovery. Using an endothelin-1-induced focal permanent stroke model, Cheng et al. reported that dl-NBP enhanced the remyelination process and increased white matter integrity by promoting differentiation and maturation of oligodendrocyte precursor cells in peri-lesional white matter (Cheng et al., 2019). However, it is not clear whether dl-NBP could promote white matter integrity after transient focal ischemic stroke.

In the present study, using a mouse model for transient focal middle cerebral artery occlusion (MCAO), we aim to investigate the effect as the underlying mechanism of dl-NBP on functional recovery after ischemic stroke in mice.

## Materials and Methods

### Animals

C57BL/6 mice (8–10 weeks old) were ordered from SLAC Company (Shanghai, China). Five mice were housed in one cage under a vivarium with a constant temperature (23 ± 1°C) and controlled light (12-h light/12-h dark cycle). They were given free access to food and water. The University Committee on Animal Care of Soochow University approved the animal procedures, which were performed according to the NIH Guide for the Care and Use of Laboratory Animals. Every effort was made to minimize animal suffering and to reduce the number of animals.

### Focal Cerebral Ischemia and Reperfusion Model in Mice

Middle cerebral artery occlusion (MCAO) was performed by an intraluminal monofilament technique (Gu et al., 2012). Briefly, mice were anesthetized with isoflurane (4% for induction and 1.75% for maintenance) in N_2_:O_2_ (70%:30%) under spontaneous respiration during surgical procedures, and the body temperature was maintained at 37.5°C ± 0.5°C using a heating pad. After a midline neck incision, the right common and external carotid arteries were isolated and ligated under microscope. A 6–0 nylon monofilament thread with a silicon-coated tip was inserted into the right internal carotid artery through the common carotid artery. The thread was advanced until it reached the origin of MCA. After 60 min of MCAO, the thread was removed to allow for reperfusion. After completion of the surgical procedures, the incision was sutured, and the mice were placed in a controlled temperature condition (24–25°C) to recover from anesthesia.

### Drug Administration and Experimental Groups

**Figure 1:**
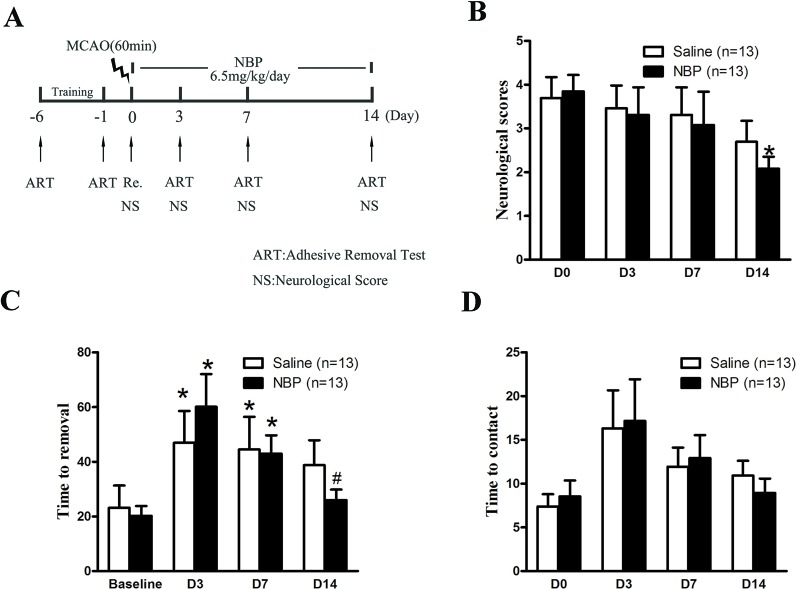
Dl-NBP treatment promoted neurological functional recovery after focal transient ischemic stroke in mice. **(A)** Outline of experimental design. Mice were subjected to 60-min ischemia and 14-day reperfusion. **(B)** Neurological scores were assessed on day 0, 3, 7, and 14. *P < 0.05 vs Saline group. **(C**, **D)** Sensorimotor functions were evaluated by the adhesive removal test. The latencies to remove **(C)** and contact **(D)** the tape were recorded. Adhesive removal tests were assessed on day 3, 7, and 14. *P < 0.05 vs baseline and ^#^P < 0.05 vs Saline group. Data are mean ± SEM, n = 13 per group.

Male had 60 min MCAO and 14 days of reperfusion. Immediately after the suture was removed, dl-NBP (6.5 mg/kg) or saline was intraperitoneally injected, and this was administered twice a day for 14 days. The neurological functional recovery after stroke in mice was detected by a tape adhesion removal test and neurological scores. The details of the experimental procedure can be found in **Figure 1A**.

### Behavioral Test

#### Neurological Score

Based on Longa’s scoring method for neurologic function deficits, the animals were scored at 24 h after they awoke from anesthetization, and symptoms of the neurologic function deficits were recorded. The scoring criteria were 0 points for no nerve injury symptom, 1 point for inability to extend the contralateral forelimb, 2 points for contralateral forelimb flexion, 3 points for mild rotation to the opposite side, 4 points for severe rotation to the opposite side, and 5 points for falling to the opposite side (Zhao et al., 2018).

#### Tape Adhesion Removal Experiment

(1) The mice were placed in a transparent box of 25 cm × 9 cm × 15 cm for 1 min. (2) Grasping the back skin of the mouse, the mouse was fixed, and a 6 mm diameter of blue circular tape (purchased from a stationery shop), which was prepared in advance, was attached to the left and right forelimbs of the mice without the hair covering the mouse. The adhesion of the tape should be controlled, keeping the adhesion of the tape on the left and right limbs consistent. (3) The tape-adhered mice were placed back in a transparent box; the time taken for the mice to tear off the tape was recorded with a video camera, and the recording time was 2 min. If a mouse did not remove the tape within the specified time, the mouse was returned to the cage and retested after 5 minutes. (4) Mice were trained once a day for 6 days before performing MCAO surgery. On day 6, the test was performed, and the experimental baseline of the left and right limb contact tape/tear tape was determined. After 3, 7, and 14 days after MACO, the mice were tested for neurological recovery.

Rating criteria:

In terms of tape contact time, from the time the mouse was placed in a transparent box and until the mouse felt the tape, the mouse appeared to swing the forelimb or tear the bit of tape with its mouth (if the mouse felt the tape, it was recorded as 120s). (2) In terms of tape removal time, from the time that the mouse was put into the transparent box and until the tape was removed from the forelimb of the mouse (if the mouse did not remove the tape, it is recorded as 120 s).

### Immunostaining for RECA-1, HIF-1α, VEGF, Dll-4, Notch, CC1, and MBP

As we described previously, the 20-μm-thick brain slice was fixed with 4% paraformaldehyde (PFA) for further analysis (Shen et al., 2018). In brief, blocking buffer (0.3% Triton X-100 and 10% normal goat serum) was applied for 2 h to block nonspecific binding and primary antibody of HIF-1α (1:100; Novus), VEGF (1:100; Abcam), Dll4 (1:50; Abcam), RECA-1 (1:50; Abcam), Notch (1:50; Cell Signaling Technology), CC1 (1:100; Abcam), and MBP (1:200; Cell Signaling Technology) were incubated overnight at 4°C. Sections were incubated with 488- (1:800, Life Technology) or Cy3-conjugated (1:800, KPL) secondary antibody for 2 h at room temperature. Confocal images were obtained using a laser scanning confocal microscope (Zeiss LSM 700, Carl Zeiss).

### Western Blot Analysis for HIF-1α, VEGF, Dll-4, Notch, and Occludin

The ischemic (I) and non-ischemic (NI) hemispheres were harvested at 14 day after MCAO (Yang et al., 2018). Tissue samples were homogenized in a lysis buffer containing protease inhibitor cocktails and phosphatase inhibitor cocktails. After homogenization, tissue samples were lysed for 1 h and then centrifuged at 14,000 g for 15 min at 4°C. Supernatants were collected and protein concentrations were detected using a BCA protein assay kit (Beyotime, China). Proteins (20 μg of total protein) were boiled and then electrophoresed in 10% or 12% SDS-PAGE acrylamide gels, transferred onto a PVDF membrane (Millipore, Billerica, MA, USA), and incubated for 2 h in TBST (Tris-buffered saline and 0.1% Tween-20) containing 5% nonfat milk. Membranes were then incubated with primary antibodies against HIF-1α (1:1000; Novus), VEGF (1:1000; Abcam), Dll4 (1:1000; Abcam), Notch (1:1000; Cell Signaling Technology), or occludin (1:300; Invitrogen) overnight at 4°C, washed in TBS-T, and then incubated with corresponding HRP-conjugated anti-rabbit or anti-mouse antibodies (1:3000; Boster, China) for 2 h at room temperature. The protein bands were developed with an enhanced luminescence reagent (Millipore) and photographed with ChemiDoc XRS+ (Bio-Rad, Hercules, CA, USA). Protein band intensities were quantified after normalization to β-actin or GAPDH and expressed as the ratios of target proteins/β-actin or GAPDH.

### Statistical Analysis

The data is expressed as mean ± SEM. Statistical analysis was carried out using one-way ANOVA with the SPSS 17.0 software. Differences were considered statistically significant when *P* < 0.05. All statistical procedures were carried out using GraphPad Prism software version 5.0 (La Jolla, CA, USA).

## Results

### Dl-NBP Promoted Neurological Functional Recovery After Focal Transient Ischemic Stroke

Dl-NBP has been shown to promote neurological functional recovery in stroke rats in an endothelin-1-induced focal permanent ischemic stroke model (Sun et al., 2017b). Here, we examined the effect of dl-NBP on neurological functional recovery in a focal transient ischemic stroke model. Neurological scores were assessed on day 1, 3, 7, and 14 after MCAO. Our results showed that dl-NBP significantly decreased the scores (~22% decrease, **Figure 1B**, *P < 0.05 vs. the saline group), suggesting that dl-NBP significantly promoted neurological functional recovery after 60-min ischemia and 14-day reperfusion.

The adhesive removal test is a sensitive method to assess sensorimotor deficits in mice (Bouet et al., 2009) and has been used to assess neurological function after stroke (Ma et al., 2018). Our results showed that, following the experimental stroke, the time to remove the tape from the affected forelimb was significantly increased (*P < 0.05 compared to baseline, **Figure 1C**), and dl-NBP treatment significantly decreased the time (~30% decrease, ^#^*P* < 0.05 compared to saline group, **Figure 1C**). In addition, the time taken for the animals to touch the adhesive tape on their affected forelimb significant increased (*P < 0.05 compared to baseline, **Figure 1D**). Treatment with dl-NBP enhanced the functional recovery, although this was not statistically significant (**Figure 1D**).

### Dl-NBP Treatment Promoted the Integrity of White Matter After Focal Transient Ischemic Stroke

**Figure 2:**
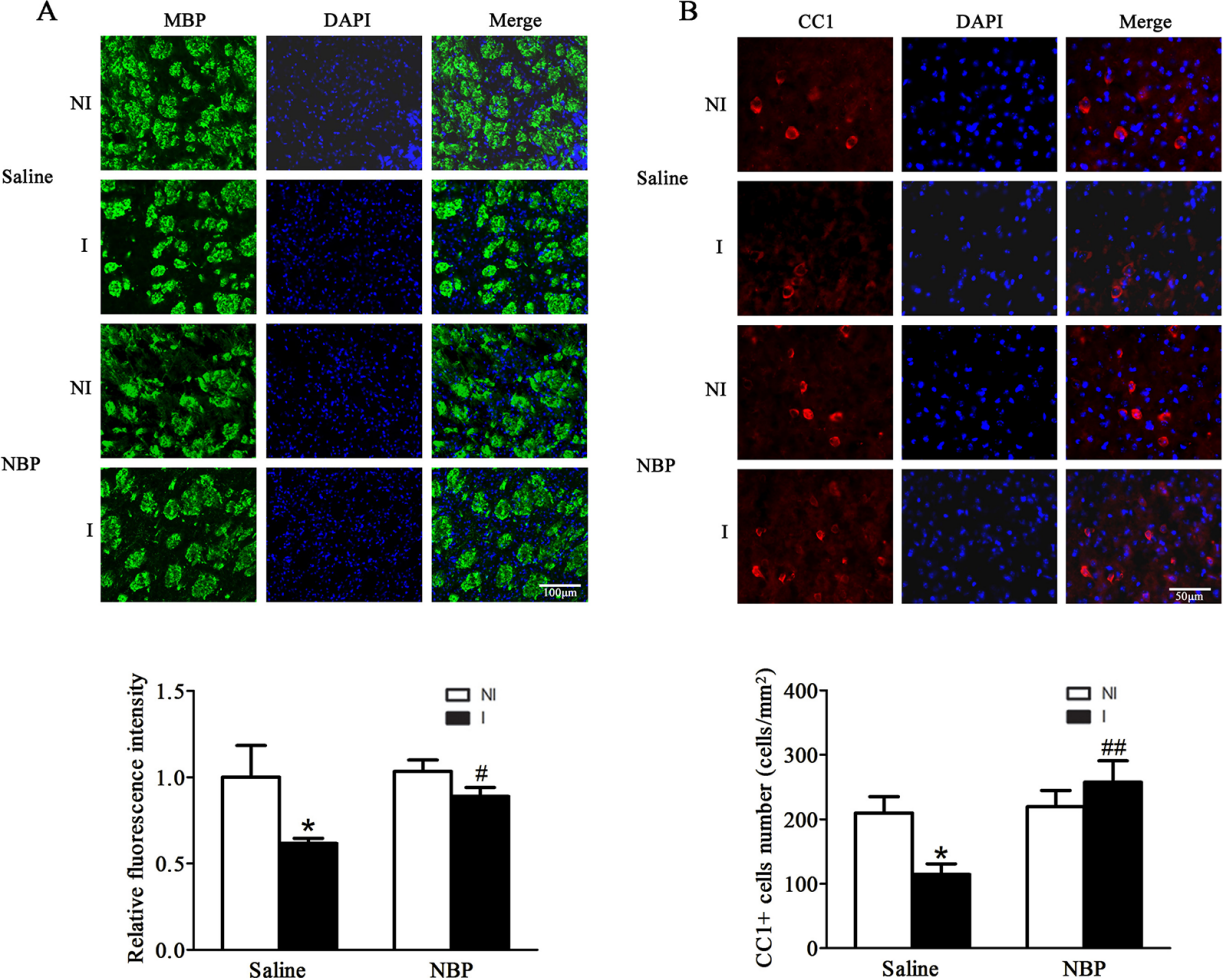
Dl-NBP treatment enhanced the integrity of white matter after focal transient ischemic stroke in mice. **(A)** Representative photomicrographs of fluorescent staining of MBP (upper panel). The relative immunofluorescence intensities of MBP were quantitated and dl-NBP significantly increased the white matter integrity (lower panel). *P < 0.05 versus NI hemisphere in Saline group. Treatment with dl-NBP significantly inhibited I/R-induced decrease. ^#^*P* < 0.05 versus Saline group. N = 3 for each group, scale bar = 100 μm. **(B)** Representative photomicrographs of fluorescent staining of CC1 (upper panel). The relative numbers of CC1+ cells were quantitated, and dl-NBP significantly increased the number of oligodendrocytes (lower panel). *P < 0.05 versus NI hemisphere in Saline group. Treatment with dl-NBP significantly inhibited I/R-induced decrease. ^#^*P* < 0.05 versus Saline group, ^##^*P* < 0.01 versus Saline group. N = 3 for each group, scale bar = 50 μm. I, ischemic hemisphere; NI, non-ischemic hemisphere.

NBP has been shown to promote remyelination and increase the integrity of white matter after endothelin-1-induced focal permanent ischemic stroke in rats (Cheng et al., 2019). Here, we examined the effect of dl-NBP on white matter integrity in a focal transient ischemic stroke model. Our results showed that dl-NBP increased the expression of myelin basic protein (MBP) (**Figure 2A**). In addition, dl-NBP increased the number of oligodendricytes as these showed more CC1 positive cells (**Figure 2B**).

### Dl-NBP Treatment Enhanced the Number of RECA-1 Positive Vessels and the Expression of Tight Junction Protein Occludin

**Figure 3:**
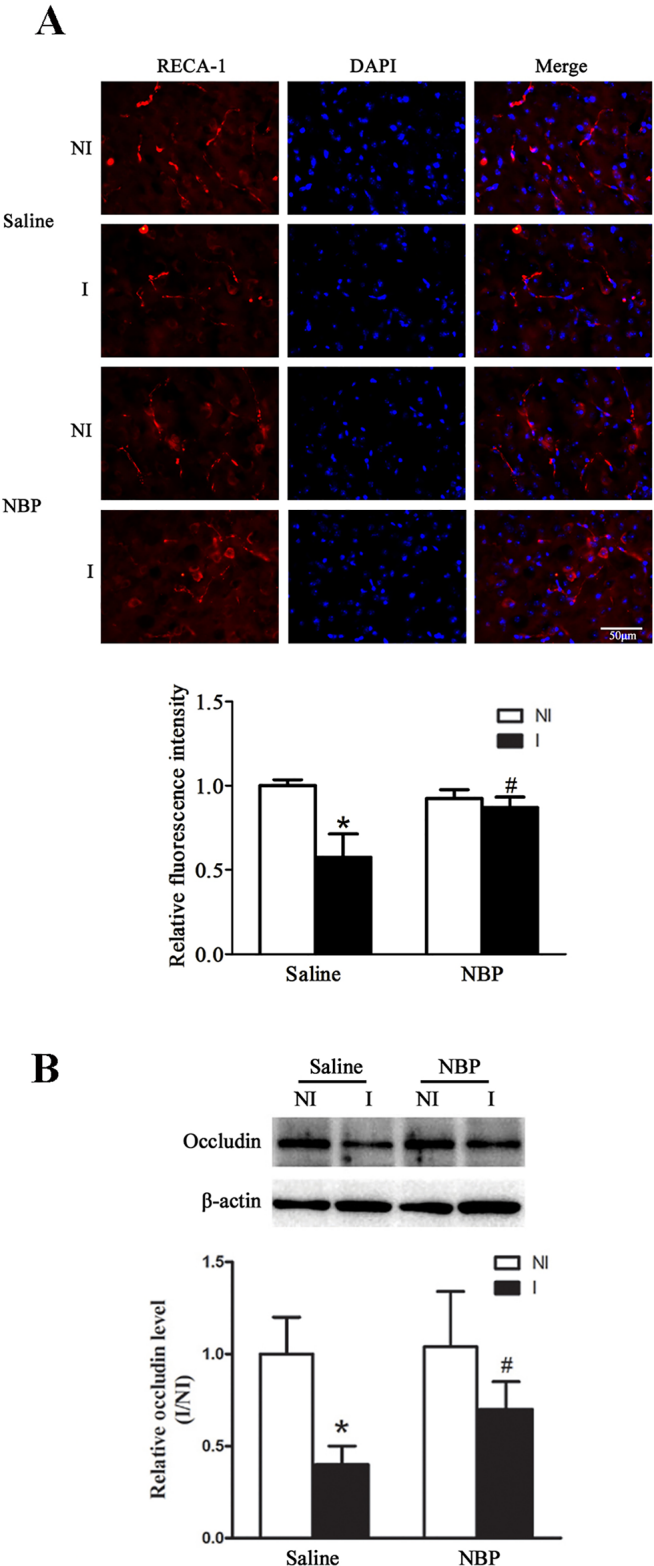
Dl-NBP treatment enhanced RECA-1 positive vessels and occludin expression after focal transient ischemic stroke in mice. **(A)** Representative photomicrographs of fluorescent staining of RECA-1 showed that dl-NBP significantly promoted the increase of the number of RECA-1 positive vessels after 60-min MCAO and 14-day reperfusion (upper panel). The relative immunofluorescence intensities of RECA-1 were quantitated (lower panel). *P < 0.05 versus NI hemisphere in Saline group. Treatment with NBP significantly inhibited this decrease. ^#^*P* < 0.05 versus Saline group. N = 3 for each group, scale bar = 50 μm. **(B)** Representative western blot revealed that I/R induced a significant decrease in occludin protein levels, and dl-NBP significantly promoted the expression of occludin. *P < 0.05 vs NI hemisphere, ^#^*P* < 0.05 vs. the Saline group, N = 6 for each group. I, ischemic hemisphere; NI, non-ischemic hemisphere.

Using stroke-prone renovascular hypertensive rats (RHRSP) to mimic hypertension, Liao et al., reported that dl-NBP treatment after a photochemical reaction-induced focal permanent MCAO model increased the number of CD31 positive vessels. This explored the effect of dl-NBP on the number of vessels after focal transient ischemic stroke. Our results showed that dl-NBP treatment significantly improved the number of RECA-1-positive vessels (**Figure 3A**, *P* < 0.05 compared to the saline group).

BBB integrity is important for functional recovery after ischemic stroke and dl-NBP has been demonstrated to alleviate ischemia-induced blood–brain barrier (BBB) damage *via* promoting the expression of tight junction proteins (Ye et al., 2019). Here, we checked the effect of dl-NBP on the expression of the tight junction protein occludin. Our results showed that dl-NBP significantly increased the expression of occludin after 60-min MCAO and 14-day reperfusion (**Figure 3B**, *P < 0.05 vs. the saline group).

### Dl-NBP Treatment Enhanced Expression of Hypoxia-Induced Factor-1α (HIF-1α) and Vascular Endothelial Growth Factor (VEGF) After Focal Transient Ischemic Stroke

**Figure 4:**
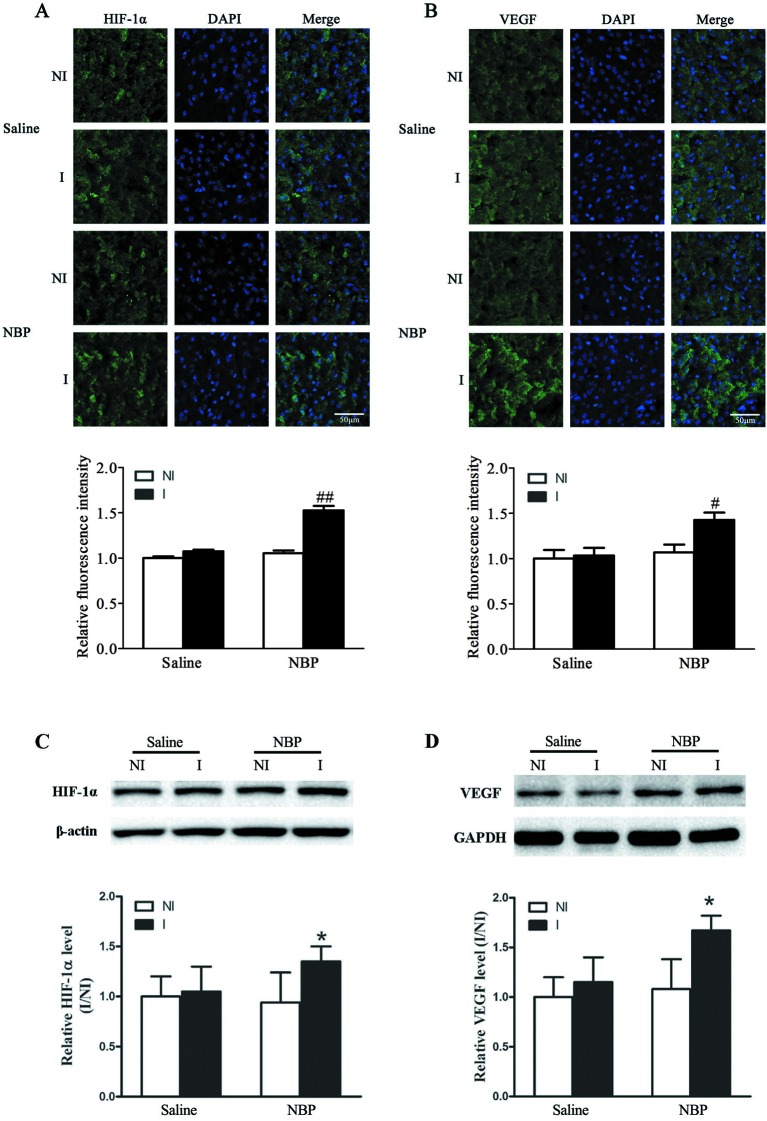
Dl-NBP increased the expression of HIF-1α and VEGF after focal transient ischemic stroke in mice. **(A**, **B)** Representative photomicrographs of fluorescent staining of HIF-1α (**A**, upper panel) and VEGF (**B**, upper panel). The relative immunofluorescence intensities of HIF-1α and VEGF were quantitated, and dl-NBP significantly increased the expression of HIF-1α (**A**, lower panel) and VEGF (**B**, lower panel). N = 3 for each group, scale bar = 50 μm. **(C**, **D)** Representative western blot showed the bands of HIF-1α (**C**, upper panel) and VEGF (**D**, upper panel). The band intensities of HIF-1α (**C**, lower panel) and VEGF (**D**, lower panel) were quantitated after normalization to the β-actin. Treatment with dl-NBP induced significant increase in the protein levels of HIF-1α (**C**, lower panel) and VEGF (**D**, lower panel). *P < 0.05 vs. the Saline group, n = 6 for each group. I, ischemic hemisphere; NI, non-ischemic hemisphere. ^#^*P* < 0.05 vs. Saline group, ^##^*P* < 0.01 vs. Saline group.

HIF-1α and VEGF have been shown to play important roles in increasing the number of blood vessels (Pajusola et al., 2005). Here, we checked the effect of dl-NBP on the expression of HIF-1α and VEGF. Our immunostaining results showed that dl-NBP treatment significantly increased the expression of HIF-1α (**Figure 4A**) and VEGF (**Figure 4B**). Western blot results confirmed the immunofluorescence results, showing that dl-NBP treatment significantly increased the expression of HIF-1α (**Figure 4C**, *P* < 0.05 vs. the saline group) and VEGF (**Figure 4D**, *P* < 0.05 vs. the saline group).

### Dl-NBP Enhanced the Expression of Notch and Delta-Like-4 (Dll4) After Focal Transient Ischemic Stroke

**Figure 5:**
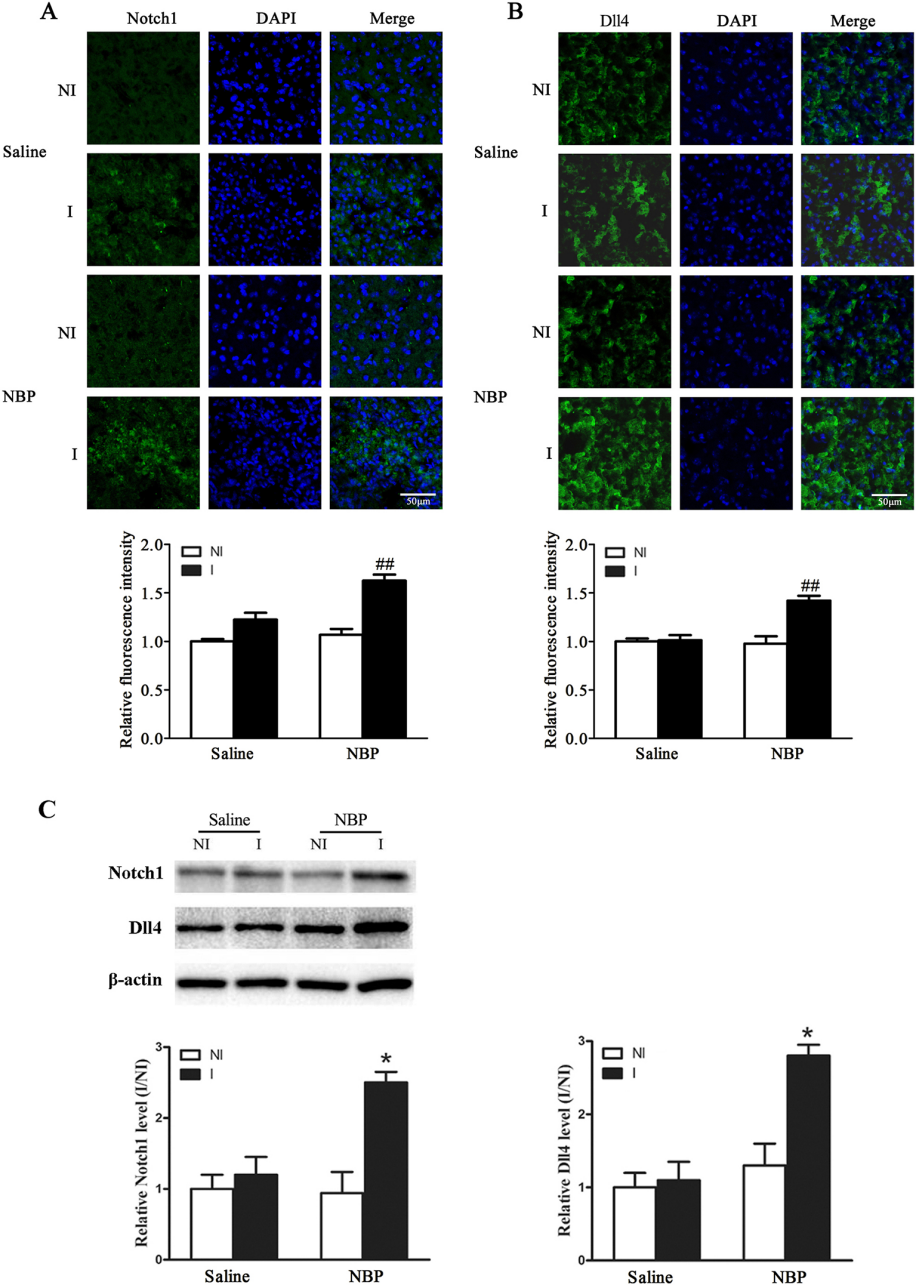
Dl-NBP increased the expression of Notch and Dll4 after focal transient ischemic stroke in mice. **(A)** Representative photomicrographs of fluorescent staining of Notch (**A**, upper panel) and Dll4 (**B**, upper panel). The relative immunofluorescence intensities of Notch and Dll4 were quantitated, and dl-NBP significantly increased the expression of Notch (**A**, lower panel) and Dll4 (**B**, upper panel). N = 3 for each group, scale bar = 50 μm. **(C)** Representative western blot revealed that the bands of Notch and Dll4 (upper panel). The band intensity of Notch and Dll4 was quantitated after normalization to the β-actin. Treatment with dl-NBP significantly increased the protein levels of Notch (lower left panel) and Dll4 (lower right panel). *P < 0.05 vs. the Saline group, n = 6 for each group. I, ischemic hemisphere; NI, non-ischemic hemisphere. ^##^*P* < 0.01 vs. Saline group.

VEGF and Dll4, a Notch ligand, emerge as the yin and yang of angiogenesis for embryonic vascular development and tumor angiogenesis (Li and Harris, 2009). Here, we investigated the expression of VEGF and Dll4 expression after focal transient ischemic stroke. Our results showed that dl-NBP significantly increased the expression of Notch (**Figure 5A**) and Dll4 (**Figure 5B**). Western blot results confirmed the immunofluorescence results, showing that dl-NBP increased the expression of Notch and Dll4 (**Figure 5C**, *P* < 0.05 vs. the saline group).

## Discussion

tPA is the only FDA-approved drug to treat acute ischemic stroke, but it is only limited to people who have received thrombolytic therapy because of a limited therapeutic time window and side effects of hemorrhage transformation (Wang et al., 2011; Wei et al., 2017). Along with looking for a strategy to increase prompt arterial recanalization by extending the tPA thrombolysis time window and endovascular treatment, it is also urgent to find a strategy to promote neurological functional recovery after acute ischemic stroke.

L-3-n-butylphthalide (l-NBP) was originally extracted from the seeds of *Apium graveolens* Linn. It has been shown to promote functional recovery after a focal transient ischemic stroke model (Yang et al., 2015); however, limited resources from plant extracts restricted its application in clinic. The racemic dl-3-n-butylphthalide (dl-NBP) was chemically synthesized, and it resolved this problem. Although dl-NBP, which was approved by the FDA of China for the treatment of acute ischemic stroke in 2002 (Wang et al., 2018), has been shown to promote neuroplasticity, motor recovery in stroke rats (Sun et al., 2017b), and enhance the remyelination process and white matter integrity using an endothelin-1 focal permanent cerebral ischemia model that could mimic those patients who have no opportunity to receive either tPA thrombolysis or endovascular therapy, it is not clear whether dl-NBP could promote functional recovery in a focal transient cerebral ischemia model that could mimic those patients who have opportunity to receive either tPA thrombolysis or endovascular therapy. Using a focal transient mice MCAO model, we collected several important findings: 1) dl-NBP treatment promoted functional recovery assessed by neurological scores and adhesive remove test; 2) dl-NBP treatment promoted white matter integrity; 3) dl-NBP treatment increased the number of microvessels and upregulated the expression of the tight junction protein occluding; and 4) dl-NBP treatment upregulated the expression of HIF-1α-VEGF and Notch-Dll4. In conclusion, our study provided evidence that dl-NBP treatment could also promote functional recovery after focal transient ischemia stroke, and this recovery is associated with upregulated white matter integrity, number of microvessels, and the tight junction protein occludin.

Previous study showed that NBP improved rats’ functional recovery following spinal cord injury (He et al., 2017), induced neuro-protection, regenerative repair, and functional recovery after traumatic brain injury in mice (Zhao et al., 2017). It also improved chronic cerebral ischemia-induced cognitive deficits in rats (Xu et al., 2012) and enhanced functional recovery in the endothelin-1-induced focal permanent stroke model (Sun et al., 2017b). Our results showed that dl-NBP improved neurological functional recovery after focal transient ischemic stroke. This result suggests that dl-NBP may promote the functional recovery for those patients who received either tPA thrombolysis or endovascular treatment.

Increasing the integrity of the white matter (Etherton et al., 2017) and the integrity of the BBB (Jiang et al., 2017) was a great contribution to neurological functional recovery. Using a endothelin-1-induced focal permanent stroke model, Cheng et al., reported that dl-NBP enhanced the remyelination process and increased white matter integrity by promoting differentiation and maturation of oligodendricytes precursor cells (Cheng et al., 2019). Our results showed that dl-NBP could increase the integrity of white matter associated with increased of numbers of oligodendricytes after focal transient ischemic stroke. This result suggests that dl-NBP could increase the integrity of white matter for those patients who received either tPA thrombolysis or endovascular treatment.

Our study showed that dl-NBP treatment increased the expression of the tight junction protein occludin, which is the important component of the blood–brain barrier (BBB). Disruption of BBB integrity is one of the key factors in the pathological damage of white matter hyperintensities burden (Li et al., 2017). This result suggests that dl-NBP may increase the integrity of white matter through increasing the integrity of the BBB *via* upregulating the expression of tight junction proteins, which is consist with a previous study showing that NBP reduced ischemia-induced BBB damage *via* upregulation of tight junction proteins (Ye et al., 2019).

Using stroke-prone renovascular hypertensive rats (RHRSP) to mimic hypertension, Liao et al., reported that dl-NBP treatment after a photochemical reaction-induced focal permanent MCAO model increased the quantity of CD31-positive vessels and upregulated expressions of HIF-1α and VEGF (Liao et al., 2009), which play important roles in angiogenesis, vasculogenesis (Leung et al., 1989), and stroke-induced BBB damage (Sun et al., 2017a; Shen et al., 2018). Our result showed that dl-NBP treatment increased the number of RECA-1 positive vessels after focal transient ischemic stroke, accompanied by upregulated HIF-1α and VEGF, suggesting that dl-NBP treatment may increase the number of RECA-1-positive vessels though upregulating HIF-1α and VEGF after focal transient ischemic stroke in non-hypertension patients.

It has been reported that VEGF induces Dll4/Notch signaling while Dll4/Notch signaling also modulates the VEGF pathway. Dll4 and VEGF emerge as the yin and yang of angiogenesis for embryonic vascular development and tumor angiogenesis (Li and Harris, 2009). Our results showed that not only HIF-1α-VEGF but also Dll4-Notch is involved in dl-NBP-induced increase of the number of RECA-1-positive vessels, suggesting that Dll4/notch and HIF-1α/VEGF may be the yin and yang of the increase in blood vessel numbers during functional recovery after focal transient ischemic stroke.

In summary, our results provide evidence that dl-NBP treatment promotes functional recovery after focal transient ischemic stroke accompanied by an increase in the integrity of white matter, expression of occludin, RECA-1-positive vessels, and upregulation of HIF-1α-VEGF and Notch-Dll4. As ischemic stroke is a complex disease involving multiple mechanisms, multi-target medication may be more powerful compared to single-target drugs. Dl-NBP has a multi-target neuroprotective effect (Huang et al., 2018), and it is therefore a promising therapeutic agent for ischemic stroke (Wang et al., 2018).

## Data Availability Statement

All datasets generated for this study are included in the article/supplementary material.

## Ethics Statement

The animal study was reviewed and approved by the University Committee on Animal Care of Soochow University.

## Author Contributions

YFS, ZL, JG, CX, SQ, XZ, BZ, YJ and YYS performed experiments, collected data, and did the statistical analyses. YFS, JG, and YW wrote the manuscript. All authors have read and approved the final manuscript.

## Funding

Grants from the Science Technology Department of Zhejiang Province (grant no. 2018C37093 and grant no. LGF20H090018) and the Health and Family Planning Commission of Zhejiang Province (grant no. 2017KY653) supported the current study.

## Conflict of Interest

The authors declare that the research was conducted in the absence of any commercial or financial relationships that could be construed as a potential conflict of interest.

## Abbreviations

BBB, blood–brain barrier; dl-NBP, dl-3-N-butylphthalide; Dll4, Delta-like ligand 4; EB, Evan’s blue dye; HIF-1α, hypoxia-induced factor-1α; I/R, ischemia/reperfusion; l-NBP, l-3-n-butylphthalide; MCAO, middle cerebral artery occlusion; MBP, myelin basic protein; TJPs, tight junction proteins; tPA, tissue plasminogen activator; VEGF, vascular endothelial growth factor.
